# WVMDA: Predicting miRNA–Disease Association Based on Weighted Voting

**DOI:** 10.3389/fgene.2021.742992

**Published:** 2021-09-29

**Authors:** Zhen-Wei Zhang, Zhen Gao, Chun-Hou Zheng, Lei Li, Su-Min Qi, Yu-Tian Wang

**Affiliations:** ^1^ School of Cyberspace Security, Qufu Normal University, Qufu, China; ^2^ School of Computer Science and Technology, Anhui University, Hefei, China

**Keywords:** miRNA-disease association, credibility similarity, weighted voting, miRNA, disease

## Abstract

An increasing number of experiments had verified that miRNA expression is related to human diseases. The miRNA expression profile may be an indicator of clinical diagnosis and provides a new direction for the prevention and treatment of complex diseases. In this work, we present a weighted voting-based model for predicting miRNA–disease association (WVMDA). To reasonably build a network of similarity, we established credibility similarity based on the reliability of known associations and used it to improve the original incomplete similarity. To eliminate noise interference as much as possible while maintaining more reliable similarity information, we developed a filter. More importantly, to ensure the fairness and efficiency of weighted voting, we focus on the design of weighting. Finally, cross-validation experiments and case studies are undertaken to verify the efficacy of the proposed model. The results showed that WVMDA could efficiently identify miRNAs associated with the disease.

## Introduction

MicroRNA (miRNA) is a class of non-coding single-stranded RNA with a length of approximately 22 nucleotides, which play a huge role in cell differentiation, biological development, and disease attack (Ambros, 2001; Lee and Ambros, 2001; Ambros, 2004; Bartel, 2004). By comparing the expression profiles of different miRNAs in cancer cells and normal cells, the researchers found that some miRNAs can inhibit the occurrence and development of malignant tumors (Esquela-Kerscher and Slack, 2006; Huang et al., 2008; Wang et al., 2013), such as breast cancer (Blenkiron et al., 1981) and prostate cancer (Garzon et al., 2006). Therefore, the discovery of disease-related miRNAs is of great significance to prevent and treat human diseases. However, it is expensive and time-consuming to look for miRNAs associated with the disease through biological experiments. Therefore, a large number of calculation methods have been developed over the past several years (Ji et al., 2020; Chen et al., 2019; Zhao et al., 2019), and some relevant datasets have also been constructed (Jiang et al., 2009; Yang et al., 2014; Yang et al., 2016; Huang et al., 2018).

In the past, a large number of methods based on measuring biological information have been established, and this idea has been the main theme of miRNA–disease association prediction. Based on the hypothesis that miRNAs with similar functions are related to the same diseases (Chen et al., 2021), Jiang et al. (2010) developed a model that uses hypergeometric distribution to determine the association between diseases and miRNAs. Since then, most researchers have based their predictions on this assumption. Yang et al. (2018) proposed a new algorithm, MiRGOFS, to measure semantic similarity and miRNA similarity based on GO terms. Chen et al. (2016) predicted potential disease-associated miRNAs by integrating miRNA functional similarity (Chen et al., 2015), disease semantic similarity, and Gaussian interaction profile kernel similarity, which is a calculation method that integrates a variety of biological information and greatly reduces the time and expenditure of biological experiments. In addition, introducing associated biological information also serves as a supplementary reference for predictive goals. Chen et al. (2018) introduced lncRNA into miRNA–disease association prediction. They constructed a miRNA–lncRNA–disease heterogeneous network and applied label propagation to identify disease-related miRNAs. Analogous to introduce other types of data, Ji et al. (2020) integrated the association between miRNA and protein and the association between protein and disease to build a tripartite network. Zheng et al. (2020) first introduced incremental learning into the field of biological association prediction. This method can distinguish the associations of previous training when adjusting new data, which strengthens the ability of acquisition, adjustment, and transfer to learning the interaction mode of miRNA and disease.

In addition to integrated biological information as a research subject, researchers also put forward a variety of colorful models, providing inspiration for follow-up research. Chen et al. (2020) presented KBMFMDA to estimate the association network by project miRNA and disease into a unified subspace. This method combines kernel-based nonlinear dimensionality reduction, matrix factorization, and binary classification. Zhao et al. presented the ABMDA to infer potential associations of miRNA–disease, which utilized a random sampling way to balance the positive and negative samples. Besides, ABMDA applied the decision tree to serve as weak classifiers that were integrated to improve the accuracy of the provided learning method. (Toprak and Eryilmaz, 2020) used weighted known nearest neighbor and network congruence projection techniques to predict new miRNA–disease relationships after integrating multiple similarity degrees. The model NCMCMDA (Chen et al., 2020) combined neighborhood constraint with matrix completion and provided a new way to predict potential associations with similarity information. After the task of recovering missing associations was transformed into an optimization problem, the model solved it with a fast iterative shrinkage threshold algorithm. SMALF (Liu et al., 2021) uses XGBoost as the final prediction model and stacked automatic encoders learn miRNA potential features and disease potential features from the original miRNA–disease association matrix, which helps to improve the sparsity and incompleteness of existing datasets.

It is worth mentioning separately that the application of neural networks provides a novel idea for predicting disease-related miRNAs. DBNMDA (Chen et al., 2020) constructed feature vectors for all miRNA–disease pairs to pretrain restricted Boltzmann machines and put the same amount of positive and negative samples into the deep-belief network to get the final prediction results. Li et al. (2021) proposed GAEMDA to identify potential miRNA–disease associations in an end-to-end manner. In multilayer perception machine learning of diverse dimensions of semantic information, the introduction of a graph neural network serves to aggregate the neighborhood information of nodes. The model NIMCGCN (Li et al., 2020) put miRNA similarity and disease similarity into the graph convolutional neural network to learn the potential feature representation of miRNA and disease, and then these features were input into the new neural induction matrix completion model to train its parameters in a supervised manner. Finally, the trained model is used to recover the unknown association.

The above methods provide us with important references, while label propagation (Chen et al., 2018; Yu et al., 2018) and the weighted voting method (Tong and Kain, 1988; Campbell and Kelly, 2010) directly give us great inspiration. In the label propagation algorithm, the elements in the adjacency matrix are iterated with the similarity matrix as a reference until the adjacency matrix converges, and the converged adjacency matrix is used to infer potential associations. Weighted voting is a method of apportioning an unequal number of votes to members of a special proportion. Referring to the algorithm of label propagation, and considering the weighted voting method, we combine the two to get the weighted voting-based model for predicting miRNA–disease association (WVMDA), which does not require iteration.

WVMDA is also a recommendation algorithm in essence, and the main factor that assesses its performance is the construction of voting weight. Its purpose is tantamount to control the weight to get the voting result as fair as possible, and not to let the members of a certain class control the whole situation, and also not to let some classes have no sense of existence. In addition to the design prediction model, we also handled similarity. First, we construct the credibility similarity and use it to complete the existing dataset. Second, we design a filtering method to extract more reliable similarity information while eliminating noise interference as much as possible. In the experimental part, we visualized the processing of similarity to observe its effect. The five-fold cross-validation (5CV) and global leave-one-out cross-validation (LOOCV) were used to measure the performance of our method, and AUC values of 0.9537 and 0.9683 were obtained, respectively. In addition, we performed case studies on human prostate tumors and looked for the top predictor miRNAs in other datasets, and the results showed that our method identified the majority of disease-related miRNAs. In conclusion, WVMDA effectively optimizes the similarity and has certain reliability in predicting miRNA–disease association.

## Materials and Methods

### Human miRNA–Disease Associations

In this paper, we downloaded the validated association of miRNA–disease from the HMDD v2.0 database. We defined an adjacency matrix $A\in R^{n\times m}$ to designate the association between miRNAs and diseases. The two dimensions of the matrix correspond to 495 miRNAs and 383 diseases, respectively, and 5,430 of the 189,585 nodes are known associations that have been verified experimentally. The adjacency matrix A was defined as:

$$\begin{array}{c} \begin{array}{c} \{\begin{array}{c} A\left(m_{i},d_{j}\right)=1\;\;\;\;\;\;\;\;\;\;\;\;\;\;\;\;\;\;miRNA\;m_{i}\;has\;association\;with\;d_{j}\\ A\left(m_{i},d_{j}\right)=0\;\;\;\;\;\;\;\;\;\;\;\;miRNA\;m_{i}\;has\;no\;association\;with\;d_{j} \end{array} \end{array} \end{array}$$ (1)

### miRNA Functional Similarity

The functional similarity of miRNAs was calculated based on the basic assumption that functionally similar miRNAs tended to be associated with similar diseases (Cui, 2010). We can load miRNA functional similarity data from http://www.cuilab.cn/files/images/cuilab/misim.zip. From these data, we constructed $FM\in R^{495\times 495}$ to account for the functional similarity of miRNA, where $FM\left(m_{i},m_{j}\right)$ represents functional similarity between miRNA $m_{i}$ and $m_{j}$ .

### Disease Semantic Similarity

The MeSH database includes many disease descriptions (Lipscomb, 2000). Directed acyclic graphs (DAG) are used to calculate disease semantic similarity. For node *D*, we define *D*(*D*) = [*T*(*D*), *E*(*D*)], where *T*(*D*) and *E*(*D*) are the nodes set and edges set, respectively. (*D*) includes node *D* and its ancestor nodes, and (*D*) represents the direct connection between parent nodes and child nodes. The contribution value of disease d to the semantic value of disease D can be calculated according to the following formula:

$$\begin{array}{c} \begin{array}{c} \{\begin{array}{c} D_{D}\left(d\right)=1\;\;\;\;\;\;\;\;\;\;\;\;\;\;\;\;\;\;\;\;\;\;\;\;\;\;\;\;\;\;\;\;\;\;\;\;\;\;\;\;\;\;\;\;\;\;\;\;\;\;\;\;\;\;\;\;\;\;\;\;\;\;\;\;\;\;\;\;\;\;\;\;\;\;\;\;\;\;\;\;\;\;\;\;\;\;\;\;\;\;if\;d=D\\ D_{D}\left(d\right)=\max\left\{\mathrm{\omega *}D_{D}\left(d^{\prime }\right)\text{|}d^{\prime }\in \mathrm{children of d}\right\}\;\;\;\;\;\;\;\;if\;d\neq D \end{array} \end{array}\; \end{array}$$ (2)

Where ω is the semantic contribution factor, and we set ω=0.5 in this paper. The setting of the contribution factor means that the contribution of *D* to itself is 1, and the contribution of other nodes to *D* will decrease as the distance increases. The semantic value of disease *D* can be defined as:

$$\begin{array}{c} V\left(D\right)=\underset{t\in T\left(D\right)}{\sum }D_{D}\left(t\right) \end{array}$$ (3)

Thus, the semantic similarity of disease $d_{i}$ and disease $d_{j}$ can be defined as follows:

$$\begin{array}{c} SD\left(d_{i},d_{j}\right)=\frac{\sum_{t\in T\left(d_{i}\right)\cap T\left(d_{j}\right)}\left(D_{d_{i}}\left(t\right)+D_{d_{j}}\left(t\right)\right)}{V\left(d_{i}\right)+V\left(d_{j}\right)} \end{array}$$ (4)

Where $SD\in R^{383\times 383}$ is the disease semantic similarity matrix composed of 383 diseases, and $SD\left(d_{i},d_{j}\right)$ is the similarity between disease $d_{i}$ and disease $d_{j}$ .

### Credibility Similarity

In order to solve the problem of the incompleteness of the existing dataset, we established a novel similarity network based on the association network in this section. The building principle is that if two miRNAs are alike in expression for the same disease, then we believe that the two miRNAs are more analogous.

**FIGURE 1:**
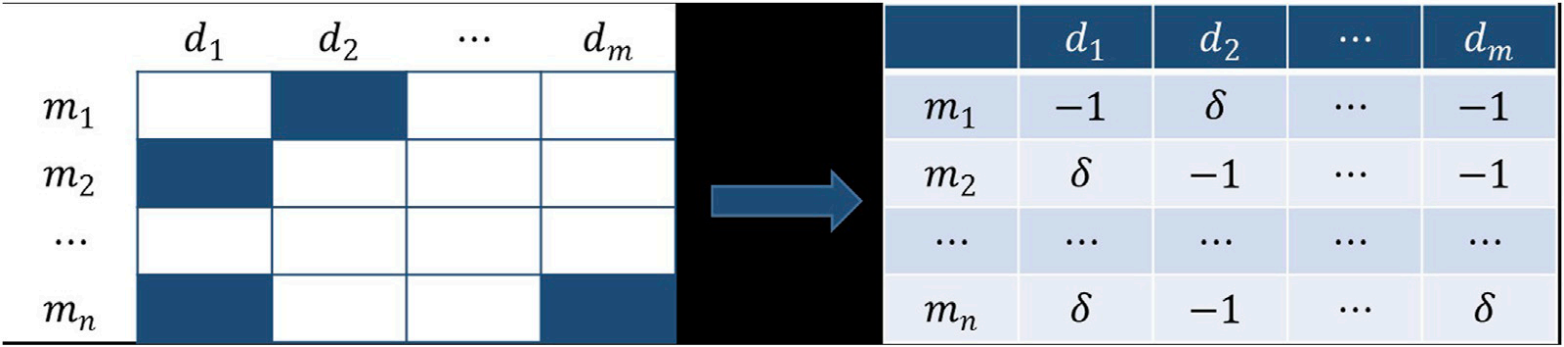
Obtain the credibility matrix C according to the incidence matrix A .

Unlike previous methods for establishing similarity, the known association matrix was first addressed. Compared with the unknown association, we thought the known association had a higher credibility. Consequently, the credibility of the known association was designed to be δ , while the credibility of the undetermined association was 1, and δ was higher than 1. Therefore, the following transformation could be performed to obtain the credibility matrix C (Figure 1).

The similarity of $m_{i}$ and $m_{j}$ can be defined as:

$$\begin{array}{c} \{\begin{array}{c} CM_{1}\left(m_{i},m_{j}\right)=C\left(m_{i}\right)\cdot C\left(m_{j}\right)\;\;\;\;\;\;\;\;\;\;\;\;\;\;\;\;\;\;\;\;\;\;\;\;\;\;\;if\;i\neq j\\ CM_{1}\left(m_{i},m_{j}\right)=0\;\;\;\;\;\;\;\;\;\;\;\;\;\;\;\;\;\;\;\;\;\;\;\;\;\;\;\;\;\;\;\;\;\;\;\;\;\;\;\;\;\;\;\;\;\;\;\;\;\;\;\;\;\;\;\;\;\;\;if\;i=j \end{array} \end{array}$$ (5)

$$\begin{array}{c} CM\left(m_{i},m_{j}\right)=\frac{\left(CM_{1}\left(m_{i},m_{j}\right)-CM_{1}{\left(m_{i}\right)}_{\min}\right)\times \left(CM_{1}\left(m_{i},m_{j}\right)-CM_{1}{\left(m_{j}\right)}_{\min}\right)}{\left(CM_{1}{\left(m_{i}\right)}_{\max}-CM_{1}{\left(m_{i}\right)}_{\min}\right)\times \left(CM_{1}{\left(m_{j}\right)}_{\max}-CM_{1}{\left(m_{j}\right)}_{\min}\right)} \end{array}$$ (6)

The range of the similarity matrix calculated according to Eq. 5 is too wide, so it is necessary to reduce it to between 0 and 1. Since our weighted voting model only uses the diagonal elements of the similarity matrix when the voters vote for themselves, its definition does not affect the result of the weighted voting, so we do not need to calculate the diagonal elements of the matrix and set them to 0. Since the elements of the principal diagonal are very large, which affects the scaling of other elements, it is also essential to set the principal diagonal element to 0 before the operation of Eq. 6. With the same method, the credibility similarity CD of disease can be constructed.

Based on miRNA functional similarity and credibility similarity of our constructs, the integrated miRNA similarity  M is built. Similarly, integrated disease similarity D can be constructed:

$$\begin{array}{c} M\left(m_{i},m_{j}\right)=\frac{\mathrm{FM}\left(m_{i},m_{j}\right)+\mathrm{CM}\left(m_{i},m_{j}\right)}{2} \end{array}$$ (7)

$$\begin{array}{c} D\left(d_{i},d_{j}\right)=\frac{\mathrm{SD}\left(d_{i},d_{j}\right)+\mathrm{CD}\left(d_{i},d_{j}\right)}{2} \end{array}$$ (8)

### WVMDA

**FIGURE 2:**
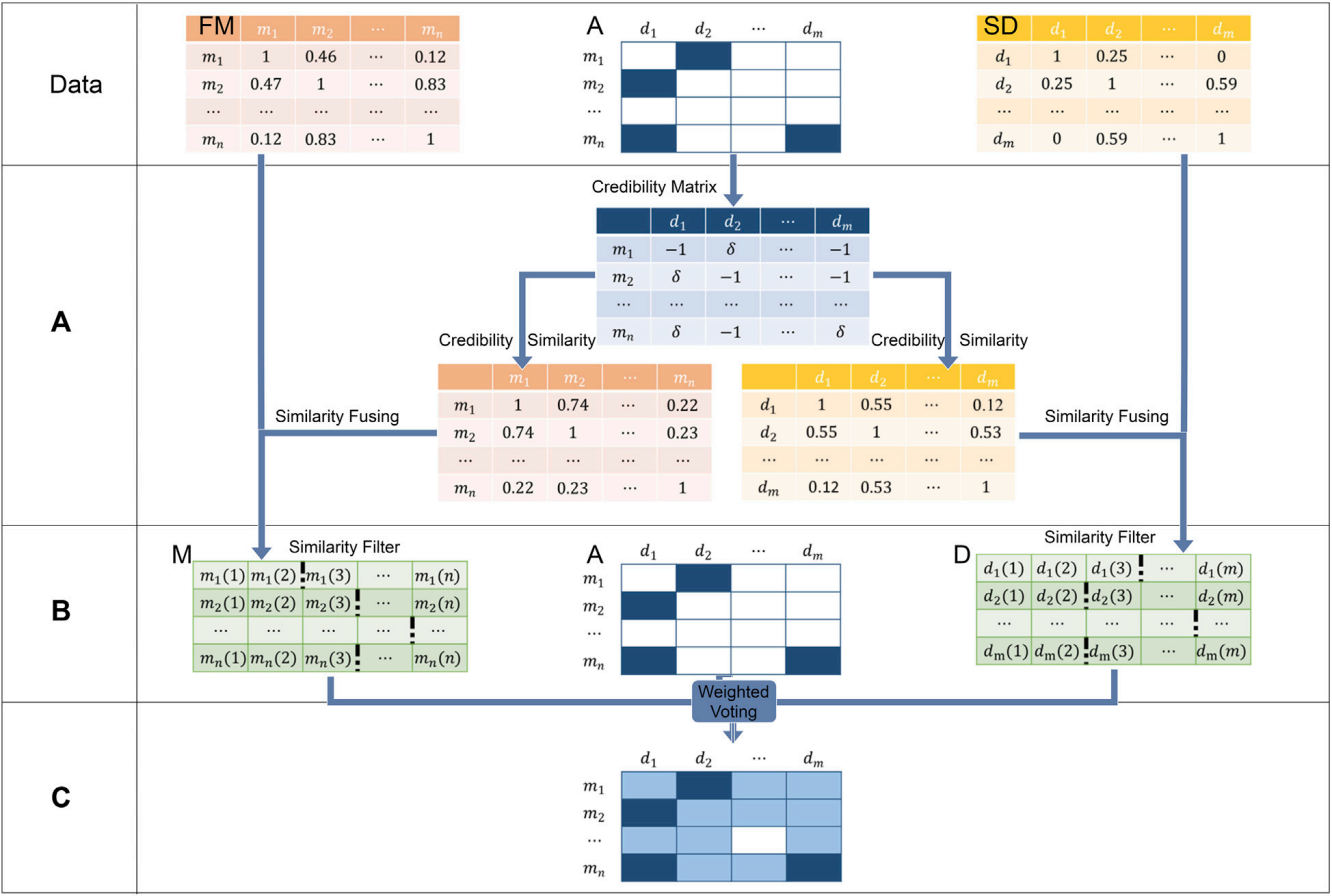
WVMDA can be divided into three steps: **(A)** we construct the credibility similarity network through known associations, **(B)** we filtered them after combining both similarity, and **(C)** weighted voting is used as the predictive model.

To infer potential associations from known miRNA–disease associations, we proposed a weighted voting method called WVMDA (Figure 2). In WVMDA, the elements of the matrix composed of diseases and miRNAs are regarded as members to be voted, where known associations are regarded as members with voting rights, and these voting members vote for other members according to the designed weight. The final voting result will serve as the prediction result, and members with higher votes are more likely to be potential associations.

#### Voting Method

**FIGURE 3:**
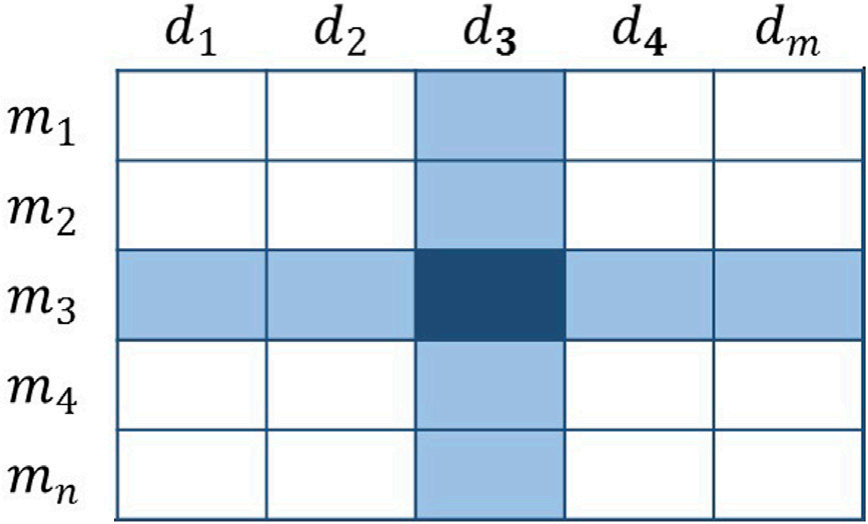
Schematic diagram of the voting method.

Assuming that there is a known association $A\left(m_{i},d_{j}\right)$ , which is regarded as a member with voting rights (Figure 3), then $A\left(m_{i}\right)$ and $A\left(d_{j}\right)$ are regarded as related groups, and all members in these two groups will receive votes from $A\left(m_{i},d_{j}\right)$ .

When voting for all candidates in group $A\left(m_{i}\right)$ :

$$\begin{array}{c} F\left(m_{i},d_{s}\right)=F\left(m_{i},d_{s}\right)+W\left(m_{i},d_{j},d_{s}\right)A\left(m_{i},d_{j}\right) \end{array}$$ (9)

where $F\left(m_{i},d_{s}\right)$ represents one candidate, $A\left(m_{i},d_{j}\right)$ represents one voter, and $W\left(m_{i},d_{j},d_{s}\right)$ represents the weight of voting from $A\left(m_{i},d_{j}\right)$ to $F\left(m_{i},d_{s}\right)$ .

When voting for all candidates in group $A\left(d_{i}\right)$ :

$$\begin{array}{c} F\left(m_{t},d_{j}\right)=F\left(m_{t},d_{j}\right)+W\left(m_{i},d_{j},m_{t}\right)A\left(m_{i},d_{j}\right) \end{array}$$ (10)

According to this idea, the final voting result can be achieved by repeating the operation on all members with voting rights. Our design of voting method is so simple, but planning its weight is the highest priority, which directly determines the rationality and effectiveness of our method.

Assuming the weight of the vote is 1, there will be some unreasonable problems. If there is only a single voter $A\left(m_{i},d_{j}\right)$ , all elements in $A\left(m_{i}\right)$ and $A\left(d_{j}\right)$ will become 1 after the voting ends. In this case, the voter and the candidate have the same status, but the voter should have a higher status as a known connection. In addition, the difference between the candidates cannot be evaluated in this case.

Furthermore, when there are several voters whose right to vote is 1, the number of votes obtained by the members of the group with the most voters will be significant. On the contrary, members of smaller groups will receive very few votes. Even though candidates with more voters are more likely to be potential association, we do not wish to see such an extreme imbalance. In this situation of extreme imbalance, some groups control the whole situation, whereas others have no meaning of existence.

#### Basic Voting Weight

**FIGURE 4:**
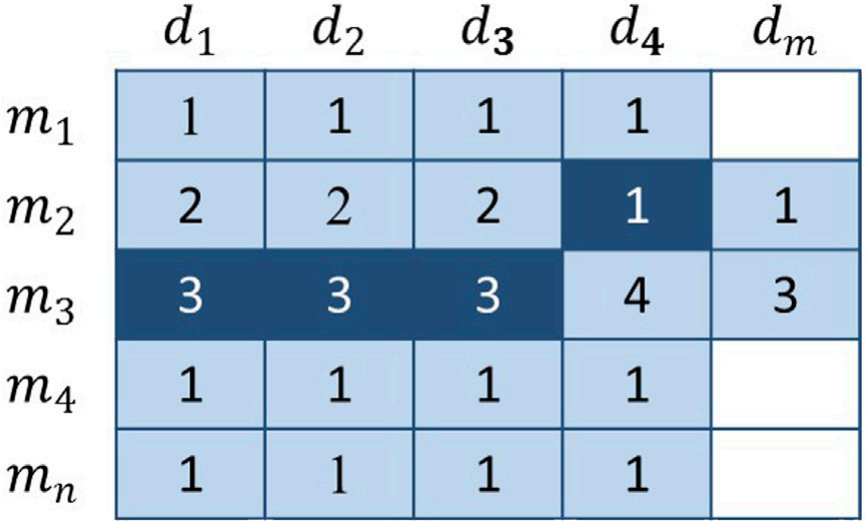
The dark-colored elements represent the voters, and the remaining elements are the candidates. The number in the figure represents the number of times the current member has been voted and does not represent the final score.

With regard to Figure 4, the vote is extremely imbalanced. Obviously, several members have the right to vote in row 3, whereas a single member has the right to vote in row 2. As a result, members of the third row will receive more votes than the second row. For example, $A\left(m_{3},d_{1}\right)$ will get three votes, and $A\left(m_{2},d_{4}\right)$ will only have one vote. As voters, it is unfair that they get so much difference in the number of votes. Furthermore, some members who have no right to vote get more votes than those who have the right to vote. For example, $A\left(m_{3},d_{4}\right)$ gets four votes and $A\left(m_{2},d_{4}\right)$ gets one vote, which is obviously not feasible. More commonly, although some members of the $A\left(m_{2}\right)$ group may be potentially association, they have far fewer votes than the members of group $A\left(m_{3}\right)$ . The right to vote should be fairly distributed, and certain groups should not be allowed to monopolize seats, nor should the votes of some groups be negligible. Based on this, we designed the basic voting weight to eliminate this gap:

$$\begin{array}{c} W_{b}\left(m_{i},d_{j},d_{s}\right)=\frac{1}{N_{\mathrm{mi}}+N_{\mathrm{ds}}-A\left(m_{i},d_{s}\right)} \end{array}$$ (11)

where $\;N_{mi}=\overset{n}{\underset{\mu =1}{\sum }}A\left(m_{i},d_{\mu }\right)$ represents the number of voting members in group $m_{i}$ ; $\;N_{ds}=\overset{m}{\underset{\nu =1}{\sum }}A\left(m_{\nu },d_{s}\right)$ represents the number of voting members in group $d_{s}$ . According to the same principle, there can be the following definitions:

$$\begin{array}{c} W_{b}\left(m_{i},d_{j},m_{t}\right)=\frac{1}{N_{\mathrm{mt}}+N_{\mathrm{dj}}-A\left(m_{t},d_{j}\right)} \end{array}$$ (12)

The basic voting weight is determined by the number of voters linked to the current candidate. This design ensures that the overall number of votes for each candidate is 1. Because the basic voting weight reduces the voting rights of voters from related groups, each group will not be dominant or trivial. However, this kind of operation will make it impossible to reflect the differences of all potential connections, so we need to further plan the uniqueness of each vote.

#### Group Weight

Following the above ideas, we have successfully assigned the weight of each vote that guarantees that the total number of votes for each candidate is 1. But common sense is that the bigger the group, the bigger their voice, meaning that the current candidate should be more credible if recommended by more voters. Therefore, we must consider extending the influence of larger groups a little more. We design weights according to the influence of the group to improve the voice of the group:

$$\begin{array}{c} W_{g}\left(m_{i},d_{j},d_{s}\right)=N_{\mathrm{mi}}^{e} \end{array}$$ (13)

$$\begin{array}{c} W_{g}\left(m_{i},d_{j},m_{t}\right)=N_{\mathrm{dj}}^{e} \end{array}$$ (14)

where $N_{mi}$ represents the number of voting members in group $m_{i}$ , and  e is utilized to control the size of the group weight. The bigger the e , the more obvious the power difference of the group will be. In order not to allow certain groups to dominate, we should design the e to be slightly smaller. In this experiment, we set e to be one-third. Due to the design of the group weight, the gap between the candidates has begun to emerge, and the candidates in the larger teams will be in a more advantageous position.

#### Candidate’s Weight

In order to account for the different status between the voter and the candidate, as well as the difference between the different candidates, we need a reference to control the weight of the acceptance of the candidate of the vote.

Both miRNA similarity and disease similarity are generally set between 0 and 1. The higher the value, the more similar the two diseases or two miRNAs. The similarity between voters and candidates is equivalent to the efficiency of the candidate in accepting votes. Due to the difference in similarity, the efficiency of different candidates for voting is also different, which reflects the uniqueness of different candidates to a certain extent. Since the main diagonal element of the similarity matrix is 1, the absolute status of voters can also be guaranteed.

Since there is a great amount of noise in the similarity network, which affects the predictive performance of the model, we designed a filter to retain the more reliable information. Taking a row of the matrix as an example, our goal is to find the smallest valuable element in the sequence. If the sequence is arranged in descending order, the above problem is approximately to find the range that falls faster and is relatively early in the sequence. Considering the particularity of some sequences and for easier implementation of operations, we preset a hyperparameter to represent the hypothetical position and find the most reasonable element with the same level of element size as that of the hypothetical position.

**FIGURE 5:**
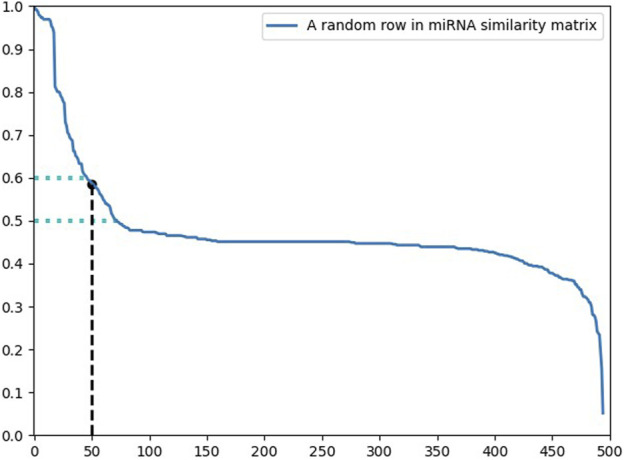
The distribution of a similarity sequence.

Figure 5 displays the distribution of a row in the miRNA similarity matrix. Due to the different number of miRNA sequences and disease sequences, it is not reasonable to use a fixed number to represent the hypothetical position. It is a better choice to use the ratio of the hypothetical position to the sequence length. In the processing of miRNA similarity, the hypothetical position was defined as $p_{h}=r*n$ , while in the processing of disease similarity, it was defined as r∗m . Assuming that the hypothetical position $p_{h}$ is 1/10th of the sequence length, the interval of its level is found. In this experiment, it is enough to divide the level with the interval of 0.1. The point in the figure above can be identified as between 0.5 and point 0.6. We define the element closest to 0.6 as the leading point $p_{l}$ and the element closest to 0.5 as the following point $p_{f}$ . For the confirmation of the final position, we followed the following principles:

$$\begin{array}{c} p=\{\begin{array}{c} p_{f},\;\;\;\;\;\;\;\;\;\;\;\;p_{f}<2p_{h}\;\;\;\;\;\;\;\;\;\;\;\;\;\;\;\;\;\;\;\;\;\;\;\;\;\;\\ p_{l},\;\;\;\;\;\;\;\;\;\;\;\;\;\;\;\;\;\;\;\;p_{f}>2p_{h}\;and\;p_{l}>\frac{p_{h}}{2}\\ p_{h},\;\;\;\;\;\;\;\;\;\;\;\;\;\;\;\;\;\;\;\;p_{f}>2p_{h}\;and\;p_{l}<\frac{p_{h}}{2} \end{array} \end{array}$$ (15)

**FIGURE 6:**
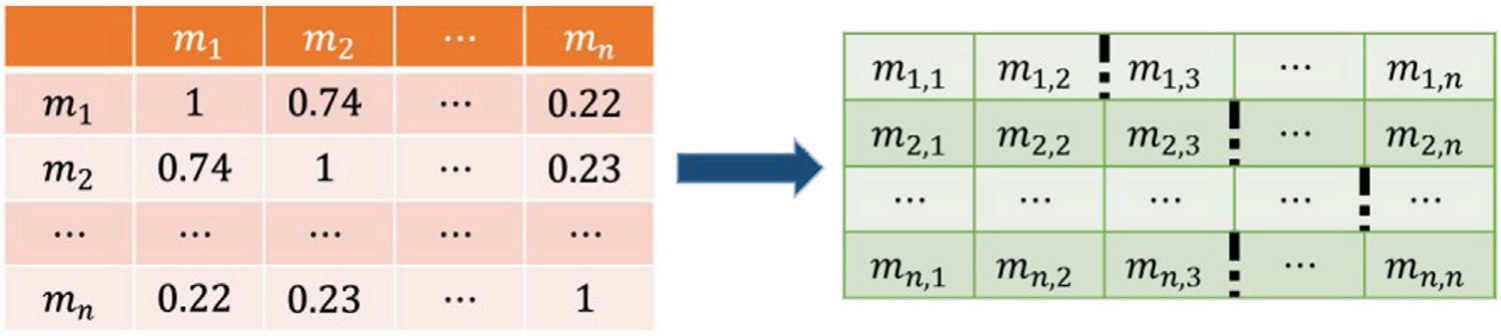
According to the p -value of each similarity sequence, the valuable information corresponding to it is retained.

Elements before the imaginary position are preserved, and elements after that are set to 0 (Figure 6). Filter each row of the matrix according to the above principles, thereby retaining an appropriate amount of reliable information depending on their different distribution.

This weight maintains the status of voters and reflects the differences between different candidates. The more scattered the elements in the similarity matrix are, the more obvious this difference is. The more reasonable the similarity matrix is constructed, the better the effect of the model.

In conclusion, we finally determined the voting weight:

$$\begin{array}{c} W\left(m_{i},d_{j},d_{s}\right)=\frac{N_{\mathrm{mi}}^{e}\times D\left(d_{j},d_{s}\right)}{N_{\mathrm{mi}}+N_{\mathrm{ds}}-A\left(m_{i},d_{s}\right)} \end{array}$$ (16)

$$\begin{array}{c} W\left(m_{i},d_{j},m_{t}\right)=\frac{N_{\mathrm{dj}}^{e}\times M\left(m_{i},m_{t}\right)}{N_{\mathrm{mt}}+N_{\mathrm{dj}}-A\left(m_{t},d_{j}\right)} \end{array}$$ (17)

The association prediction score between disease $m_{i}$ and miRNA $d_{j}$ can be defined as follows:

$$\begin{array}{c} F\left(m_{i},d_{j}\right)=\overset{m}{\underset{s=1}{\sum }}\frac{N_{\mathrm{mi}}^{e}\times D(d_{j},d_{s})\times A(m_{i},d_{s})}{N_{\mathrm{mi}}+N_{\mathrm{ds}}-A(m_{i},d_{s})}+\overset{n}{\underset{t=1}{\sum }}\frac{N_{\mathrm{dj}}^{e}\times M(m_{i},m_{t})\times A(m_{t},d_{j})}{N_{\mathrm{mt}}+N_{\mathrm{dj}}-A(m_{t},d_{j})} \end{array}$$ (18)

## Results

In this section, we conducted a number of different experiments to observe and evaluate the effectiveness of this approach, including visualization of similarity processing, adjustment of important hyperparameters, comparison with other existing methods, and analysis based on disease cases.

### Visualization of Similarity Processing

**FIGURE 7:**
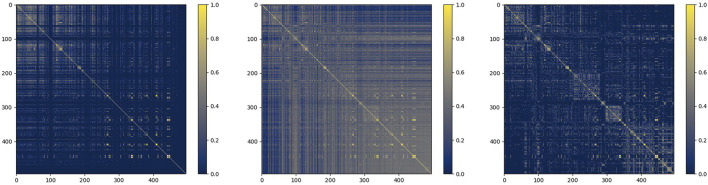
miRNA function similarity, similarity after fusion, and similarity after filtering.

Taking miRNA similarity as an example, we firstly fused functional similarity and credibility similarity, and then put it into the similarity filter. To observe the situation of similarity matrix more intuitively, we choose the heat map to express them. The process of their change is shown in Figure 7 below:

As shown in the figure, the similarity network after fusion is denser, and the similarity after filtering retains more reliable information, which provides a guarantee for the subsequent prediction.

### Performance Evaluation

In this part, we evaluate the performance of the model. The main measurement methods are 5CV and global LOOCV. 5CV uses four-fifths of the positive samples as the training set, the remaining one-fifth of the positive samples and all the negative samples as the test set, and measures the effect of the model according to the accuracy of the test. When describing its accuracy, we mainly use AUC as the measurement index. It is worth mentioning that AUC is not sensitive to whether the sample category is balanced, which is also a reason why AUC is usually used to evaluate the performance of classifier for unbalanced samples. AUC is defined as the area below the ROC curve. Among them, the ROC curve is plotted with the true positive rate (TPR) as the vertical axis and the false positive rate (FPR) as the horizontal axis. By adjusting the threshold, the probability is converted to the category, so that the TPR and the FPR are plotted as points, and the ROC curve is obtained. The calculation methods of FPR and TPR are as follows:

$$\begin{array}{c} TPR=\frac{\mathrm{TP}}{\mathrm{TP}+\mathrm{FN}} \end{array}$$ (19)

$$\begin{array}{c} FPR=\frac{\mathrm{FP}}{\mathrm{FP}+\mathrm{TN}} \end{array}$$ (20)

Among them, TP are samples truly positive and predicted to be positive, and FN are samples truly positive and predicted to be negative. Where FP are samples truly negative but predicted to be positive, TN are the samples truly positive but predicted to be negative. The ROC curve is generally above y=x . For random distribution, the ROC curve is close to y=x , so the AUC value is close to 0.5 generally. If the AUC is moving closer to 1, the better the classification effect; the closer the AUC is to 0.5, the worse the classification effect.

**FIGURE 8:**
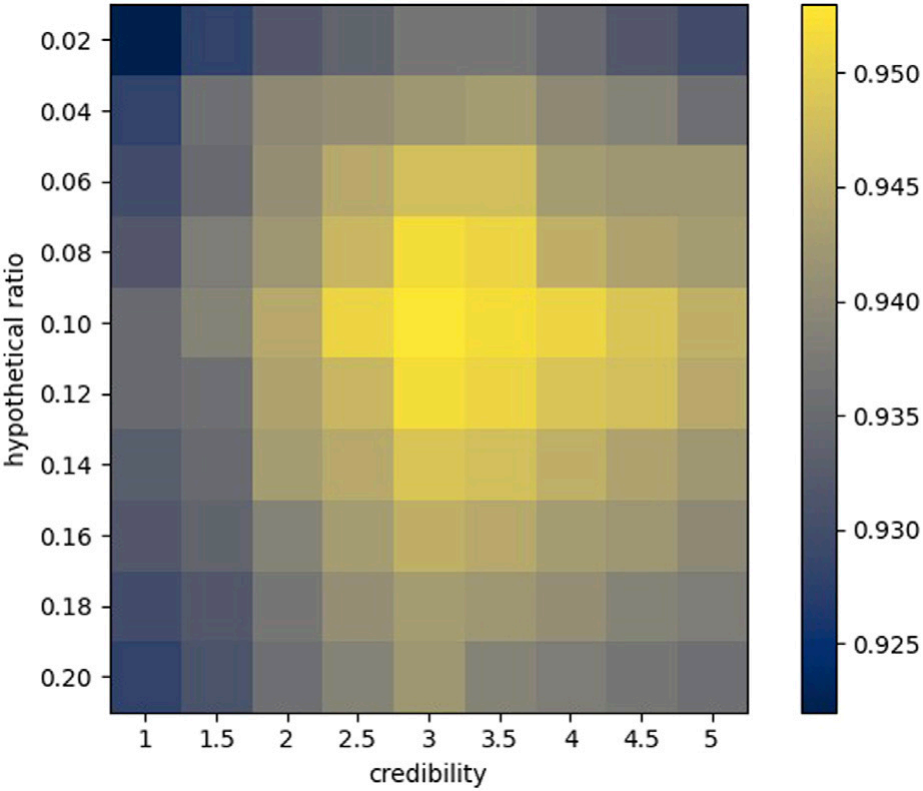
Heat map of 5CV.

According to the aforementioned indicators, we first commissioned the parameters in the model, including credibility and hypothetical position (Figure 8).

Credibility has a very significant meaning for the structure of similarity. For any two sequences $L_{1}\;=\;A\;\left(m_{i}\right)$ and $L_{2}\;=\;A\;\left(m_{j}\right)$ , the credibility similarity can be defined as $CM\left(m_{i},m_{j}\right)$ . It can be assumed that its order of magnitude is $n\delta^{2}$ , where n is the length of $L_{1}$ . If both $L_{1}\left(s\right)$ and $L_{2}\left(s\right)$ are −1, but their true values are both δ , then the similarity error is about $\frac{\delta^{2}-1}{n\delta^{2}}$ ; if one of them should be δ , the error is about $\frac{1+\delta }{n\delta^{2}}$ . If $L_{1}\left(t\right)=-1$ and $L_{2}\left(t\right)=\delta$ , but the true value of $L_{1}\left(s\right)$ is δ , the error is about $\frac{\delta^{2}+\delta }{n\delta^{2}}$ . According to the above analysis, it seems that the maximum credibility is more conducive to the establishment of similarity, but the fact is not the case: first of all, due to the relatively small number of known associations, the order of magnitude of similarity may be significantly different from $n\delta^{2}$ , which makes it impossible to blindly allow δ to take a very large number. Secondly, the larger δ is, the more obvious the role of the known association is, which goes against the original intention of making full use of all miRNA–disease connections. Based on these two points alone, it is necessary to debug δ .

The hypothetical position roughly determines the number of elements to be extracted from each similarity sequence. Due to the uniqueness of different similarity sequences, the number of optimal elements is also different. Our approach does not necessarily guarantee that the most suitable elements are extracted for each sequence, but we can approach the optimal result by adjusting the hypothetical ratio r . Multiple tests are carried out by dividing the data set for many times. The specific results are shown in Table 1.

**TABLE 1:** Prediction results under different training sets.

miRNA	1	2	3	4	5	Average
5CV	0.9485	0.9509	0.9497	0.9529	0.9481	0.9506
LOOCV	0.9639	0.9670	0.9657	0.9684	0.9621	0.9668

### Comparisons With Existing Work

**FIGURE 9:**
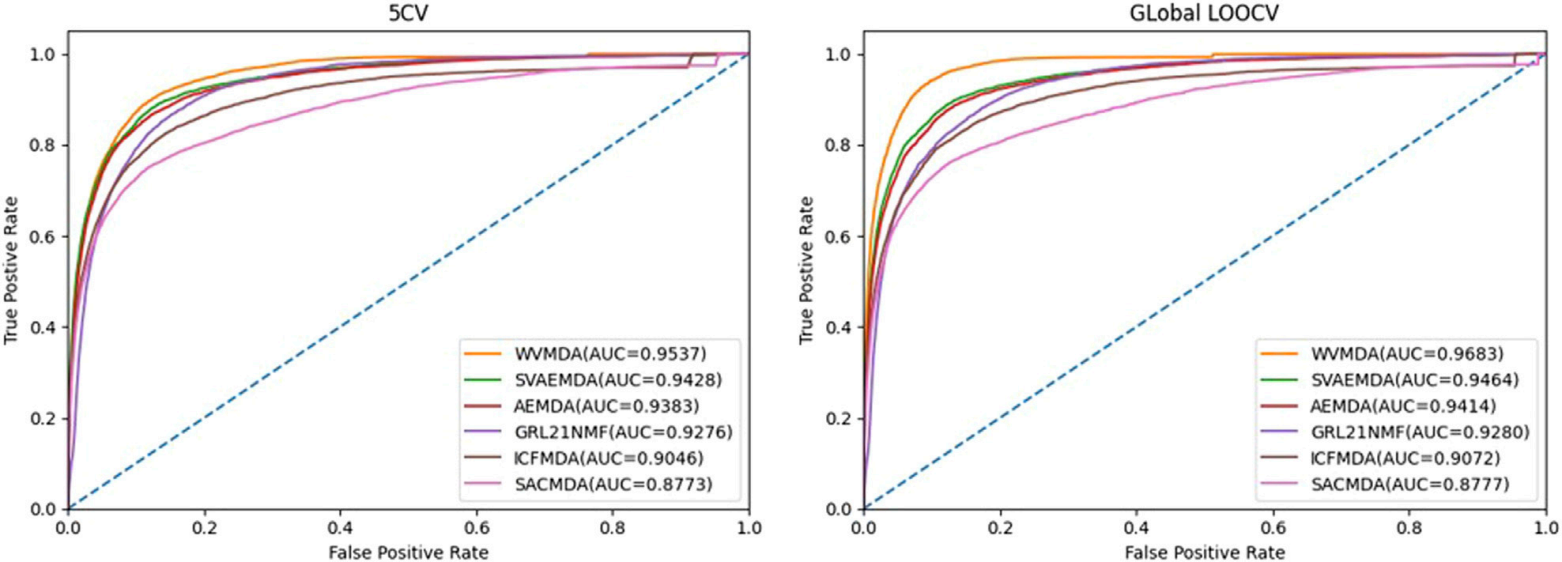
ROC curve.

In recent years, researchers have proposed many miRNA–disease association prediction methods. However, the datasets or evaluation methods used in the existing methods are not consistent. Therefore, we mainly conduct comparative experiments based on five-fold cross-validation and leave-one-out cross-validation. To confirm the validity of the WVMDA prediction results, we compared our model with the previous three models: SVAEMDA (Ji et al., 2021), ICFMDA (Jiang et al., 2018), AEMDA (Ji et al., 2020), SACMDA (Shao et al., 2018), and GRL_2, 1-NMF (Gao et al., 2020). All models were cross-validated to calculate TPR and FPR, draw the ROC curve, and calculate AUC (Figure 9). The better the performance of the model, the farther its ROC curve is from the straight line y=x , and the closer its AUC value is to 1.

It can be seen from the figure that our method is more responsive and has achieved higher AUC values, which indicates that our method has good performance.

### Case Study

In this paper, the prediction results of WVMDA were verified by taking prostate neoplasms as an example. We use the HMDD V2.0 database as the training database, and the dbDEMC 2.0 database and miR2Disease database as the validation databases, respectively, to measure the validity of the prediction model. The candidate miRNAs obtained from WVMDA were ranked according to the predicted score. If the miRNAs with the highest scores were found in the other two datasets, it would indicate that our model was effective. Finally, 28 of the top 30 miRNAs were verified in the other two datasets (Table 2). The results demonstrate the effectiveness of WVMDA in predicting unknown interactions between miRNA and disease.

**TABLE 2:** The top 30 potential miRNAs associated with prostate neoplasms.

miRNA	Evidence	miRNA	Evidence
hsa-mir-133b	D	hsa-mir-30a	M
hsa-mir-34c	D	hsa-mir-200c	D
hsa-mir-10a	D; M	hsa-let-7b	D; M
hsa-mir-29c	D	hsa-mir-182	D; M
hsa-mir-154	M	hsa-mir-155	D
hsa-mir-199a	D; M	hsa-mir-497	D
hsa-mir-330	M	hsa-mir-200b	Unconfirmed
hsa-mir-203	D	hsa-mir-373	D; M
hsa-mir-513c	M	hsa-mir-513c	M
hsa-mir-181a	D; M	hsa-mir-616	D
hsa-mir-572	D	hsa-mir-628	Unconfirmed
hsa-mir-198	D; M	hsa-mir-21	D; M
hsa-let-7d	D; M	hsa-mir-195	D; M
hsa-mir-15a	D; M	hsa-mir-371	D
hsa-mir-708	D	hsa-mir-144	D

D, dbDEMC 2.0 database; M, miR2Disease database.

### Prediction of Unknown Disease

Predicting potential miRNAs associated with unknown diseases is a huge challenge. For the convenience of experiment and verification, we selected a disease and cleared its association nodes with all miRNAs, so as to make the disease as an unknown disease. Subsequently, we put all the remaining associations into the WVMDA and observe whether its prediction results can restore the miRNAs associated with this disease. We took breast neoplasms as the case and HMDD2.0 as the database for the experiment to find out whether the 30 miRNAs with the highest prediction rank were true associations. If not, we found out whether such associations existed in other databases.

The experimental results show that 28 associations were found in the HMDD dataset, and the remaining two associations were also found in the dbDEMC dataset (Table 3).

**TABLE 3:** The top 30 potential miRNAs associated with breast neoplasms.

miRNA	Evidence	miRNA	Evidence
hsa-let-7a	H	hsa-mir-7	H
hsa-mir-141	H	hsa-mir-100	H
hsa-mir-145	H	hsa-let-7b	H
hsa-mir-10b	H	hsa-let-7d	H
hsa-mir-126	H	hsa-mir-375	H
hsa-mir-135a	H	hsa-mir-107	H
hsa-mir-151a	H	hsa-mir-34c	H
hsa-mir-182	H	hsa-mir-30d	H
hsa-mir-183	H	hsa-let-7g	H
hsa-mir-191	H	hsa-mir-302b	H
hsa-mir-200a	H	hsa-mir-320a	H
hsa-mir-200b	H	hsa-mir-625	H
hsa-mir-200c	H	hsa-mir-629	H
hsa-mir-205	H	hsa-mir-330	−H; D
hsa-mir-25	H	hsa-mir-185	−H; D

H, HMDD v2.0 database; D, dbDEMC 2.0 database.

## Discussion

The study of the possible relationship between miRNA and disease is helpful to understand the pathogenesis of disease and provide the basis for the prevention and treatment of disease. Therefore, we constructed a new miRNA–disease association prediction model based on weighted voting (WVMDA). By proposing credibility, we construct credibility similarity and use it to fill in the inadequacy of existing datasets. By designing a similarity filter, we filter the similarity to retain the reliable data and eliminate the noise. In the final weighted voting model, we mainly regulate the rationality and performance of the model based on three kinds of voting weights. It is worth mentioning that our method only needs positive samples to complete the prediction, which is very convenient for model construction and also reduces the requirements on datasets.

Under the framework of 5CV and global LOOCV, the AUC of WVMDA is 0.9537 and 0.9683, respectively, which is higher than the other methods. Furthermore, a case study on prostate neoplasm was implemented to evaluate the WVMDA model. Therefore, WVMDA can be used as a reliable biological tool for predicting potential disease-related miRNAs, and it can contribute to the discovery, prevention, and diagnosis of complex diseases. What is more, the WVMDA model still has room for improvement, and integrating more effective datasets will certainly bring great progress to future research.

## Data Availability

Publicly available datasets were analyzed in this study. This data can be found here: http://www.cuilab.cn/files/images/cuilab/misim.zip.
